# Development and feasibility of a sex- and gender-sensitive primary care intervention for patients with chronic non-cancer pain receiving long-term opioid therapy (GESCO): a study protocol

**DOI:** 10.1186/s40814-024-01564-7

**Published:** 2024-11-01

**Authors:** Christine Kersting, Johannes Just, Alexandra Piotrowski, Alexandra Schmidt, Neele Kufeld, Rebecca Bisplinghoff, Michaela Maas, Veronika Bencheva, Jordan Preuß, Birgitt Wiese, Klaus Weckbecker, Achim Mortsiefer, Petra Thürmann, on behalf of the GESCO study group, Christine Kersting, Johannes Just, Alexandra Piotrowski, Alexandra Schmidt, Neele Kufeld, Rebecca Bisplinghoff, Michaela Maas, Veronika Bencheva, Jordan Preuß, Birgitt Wiese, Klaus Weckbecker, Achim Mortsiefer, Petra Thürmann, Michaela Duck, Sven Schmiedl, Ottomar Bahrs, Thomas Cegla, Sigrid Elsenbruch, Claudia Levenig, Christina Hunger-Schoppe, Claudia Kiessling, Ulrich Klee, Ursula Lauf, Brigitte Scholz, Albine Moser, Norbert Scherbaum, Michael Specka

**Affiliations:** 1 Chair of General Practice II and Patient-Centredness in Primary Care, Institute of General Practice and Primary Care, Faculty of Health, Witten/Herdecke University, Witten, Germany; 2 Chair of General Practice I and Interprofessional Care, Institute of General Practice and Primary Care, Faculty of Health, Witten/Herdecke University, Witten, Germany; 3 Chair of Clinical Pharmacology, Faculty of Health, Witten/Herdecke University, Witten, Germany; 4 IT Services Applications, Science & Laboratory, MHH Information Technology, Hannover Medical School, Hannover, Germany; 5 Philipp Klee-Institute of Clinical Pharmacology, Helios University Hospital Wuppertal, Wuppertal, Germany

**Keywords:** Chronic pain, Opioids, Gender role, Sex, Primary care

## Abstract

**Background:**

Chronic non-cancer pain (CNCP) is a common condition worldwide. The disease burden is influenced not only by pain itself, but also by psychiatric co-morbidities, which aggravate symptoms, generally negatively influence therapies, and may thereby lead to frustration, resignation, or withdrawal. A growing body of evidence suggests that sex and gender aspects influence CNCP management as the experience of pain, the emotions associated with it, and the expression of pain may differ between women and men. In addition, doctor-patient communication is known to be influenced by gender stereotypes. Despite there being evidence on such differences, current guidelines do not consider sex- and gender-sensitive approaches. In order to examine how to adequately address the diversity of the experience and processing of pain in patients of differing sex and gender, the GESCO study aims at developing and pilot testing a sex- and gender-sensitive intervention for patients with CNCP receiving long-term opioid therapy (LTOT) in primary care.

**Methods:**

The development process is designed in accordance with the first two phases of the UK Medical Research Council. Phase I will iteratively explore, develop, and pilot the intervention’s modules using literature searches, interviews, and workshops involving stakeholders and experts. Phase II will pilot-test the novel intervention in a sample of 40 patients with CNCP under LTOT from ten general practices using an effectiveness-implementation hybrid design including a mixed-methods process evaluation focusing on implementation strategy criteria and a single-arm, pre-post comparison to determine preliminary effects in preparation for a larger effectiveness trial. The intervention will combine in-person educational sessions for general practitioners and tools to be used in patient care.

**Discussion:**

The intervention aims to improve CNCP management in primary care by empowering practitioners to reflect on their attitudes towards pain and stereotypes. Besides sex and gender aspects, awareness of other factors that might affect the care process, such as age, social conditions, or culture, is also promoted. The intention is to develop a comprehensive care concept for CNCP that considers aspects relevant for sex- and gender-sensitive care which are transferrable to other health care fields as well.

**Trial registration:**

German Clinical Trial Register DRKS00029980.

**Supplementary Information:**

The online version contains supplementary material available at 10.1186/s40814-024-01564-7.

## Background

Chronic non-cancer pain (CNCP) is a common condition worldwide, negatively affecting individuals, families, and communities as well as national economies [1–4]. Opioid therapy can significantly improve CNCP symptoms but may also pose a problem in itself [5]. Germany is among the countries with the highest per capita consumption of opioids for CNCP worldwide [6]: in 2012, 1.3% of insured persons received long-term opioid therapy (LTOT). The disease burden in patients with CNCP is influenced not only by pain itself, but also by psychiatric co-morbidities, which commonly aggravate symptoms and are known to hamper therapy [7, 8]. In everyday care, insufficient success of CNCP treatment often results in frustration for both the patient and the therapist and may lead to resignation and withdrawal on one or both sides [9, 10], frequently associated with opioid misuse [9, 11, 12].

A growing body of evidence suggests that CNCP management and co-morbidities are subject to influences associated with sex and gender aspects [13–15]. Gender is a concept considering social, environmental and situational connotations, identity and the role of a person whereas sex refers to the biological function. However, sex and gender do influence each other, which frequently impairs a differentiation between sex- or gender-specific aspects [16]. In medicine, sex and gender differences are well described for many conditions where gender differences in pain perception and coping with pain have been described in animals and humans [17–19]. In pharmacology, sex differences have been described for the pharmacokinetics as well as the pharmacodynamics of many frequently used drugs [20, 21]. Among them are opioids, where a body of evidence supports the hypothesis that gonadal hormones influence and determine sex-specific differences in pain and opioid-associated effects [22, 23].

In the context of chronic pain, sex and gender aspects are relevant not only regarding different prevalences of co-morbidities, but also in terms of experiencing and processing chronic pain itself. Among others, studies report differences regarding pain-related negative emotions such as anxiety and frustration [24], more detailed reporting of negative experiences with their physicians among women [25], and a higher prevalence of childhood trauma and family conflict in women [15]. It is also known that male and female patients with CNCP differ in how they verbally and non-verbally express pain [26] and that health care professionals’ communication is influenced by gender stereotypes [10, 27, 28].

Even though there is evidence on differences between men and women, current clinical guidelines focus on critically reviewing the therapy of patients with CNCP, examining whether opioid prescriptions are adequate and which alternatives might be used instead, but they do not consider any gender-sensitive approaches [29–32].

Despite the recognized importance of gender in doctor-patient communication, most communication skills assessment instruments in medical education neglect this factor, with only a minority incorporating gender-related content. To improve communication training for medical professionals, clearer criteria and purposes for integrating gender considerations into assessment practices are needed [33]. Doing this, it has to be taken into account that beyond the existing research on gender topics, there are still gaps in knowledge regarding the actual magnitude of gender disparities in pain care as well as on which specific interventional strategies might help to adequately address these disparities. Thus, there is a need to participatory develop more targeted group-specific care concepts for patients with CNCP on LTOT that consider a variety of sex- and gender-sensitive approaches. Such approaches should not include stereotypical standard interventions for women and men, respectively, but should increase awareness for stereotypical differences and also enable physicians to address patients in light of other individual factors such as biological, cultural or psychosocial background, which are known to be relevant for CNCP management as well [34]. As the results of dyad research suggest [35], this requires an intervention that includes elements of reflection on the physicians’ subjective attitudes towards pain and gender and their role as practitioners, in addition to improving their (sex- and gender-sensitive) communicative competencies [36]. This approach has the potential to benefit patients by fostering greater empathy and understanding in doctor-patient interactions, ultimately leading to more tailored and appropriate care.

### Objectives

The aims of the study are twofold: The first is to develop a novel gender-sensitive care for chronic non-cancer pain patients receiving long-term opioid therapy (GESCO) intervention to support individuals with CNCP receiving LTOT in primary care. In this step, the elements of the GESCO intervention including implementation strategies required to apply the intervention in the care of patients with CNCP will be developed. The second is to determine the feasibility of this intervention from the perspective of patients with CNCP and their general practitioners (GPs). This feasibility study aims to identify potential refinements to the intervention’s contents and design prior to a larger effectiveness trial, and to determine preliminary effects to estimate effect sizes for a larger trial [37, 38].

## Methods/design

**Fig. 1:**
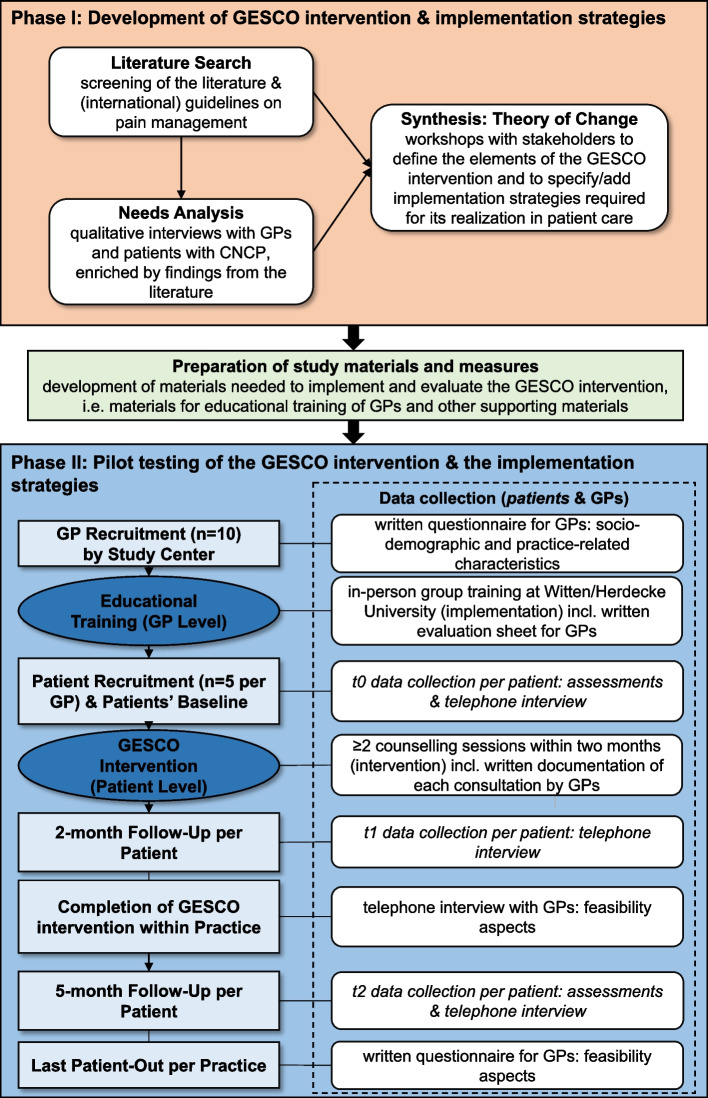
Study flow of the GESCO study

The GESCO intervention will be developed and pilot tested in terms of feasibility criteria in accordance with the two first phases of the UK Medical Research Council (MRC) Framework for developing complex interventions [39, 40]: First, a development and modeling process will be applied in order to iteratively explore, develop, and evaluate the contents and single modules of the sex- and gender-sensitive GESCO intervention by involving stakeholders and experts (phase I). Second, an exploratory mixed-methods study in a clinical sample of 40 patients with CNCP receiving LTOT, who get managed by ten primary care practices that will be educated previously, will be conducted in order to pilot-test the intervention and determine whether the intervention is feasible for a future effectiveness study (phase II) [38, 41]. The study conduct is visualized in Fig. 1.

Reporting of the study refers to the SPIRIT checklist [42], but was adapted for reporting the protocol of a feasibility study considering the CONSORT statement for pilot and feasibility trials [41, 43].

### Phase I: development of the GESCO intervention and implementation strategies

#### Study design

Starting with a theoretical phase, the existing literature will be screened for systematic reviews and original studies focusing on sex- and gender-specifics in CNCP related to pharmacological and non-pharmacological treatment, co-morbidities, patients’ needs, and physician–patient communication, as well as social dimensions. Additionally, national and international guideline recommendations for CNCP treatment will be screened to explore whether they provide any sex- and/or gender-specific recommendations. Based on the findings identified in the literature, patients with CNCP and GPs will be interviewed to assess their needs in the light of the current knowledge.

As a starting point for intervention design, the results of the literature review and stakeholder needs assessments (interviews with GPs and patients with CNCP) will be synthesized into a theory of change [44, 45]: During a participatory workshop with patients, physicians and experts (such as psychologists or sociologists), a long-term goal for the intervention will be defined. Based on the long-term goal, short and intermediate intervention outcomes, activities and inputs will be collected. In a second consecutive workshop the results from the first workshop will be specified—including strategies regarding implementation and sustainment of the intervention. Based on the theory of change, intervention materials and documents will be developed and pre-tested in general practices. The evaluation design will also follow the theory of change.

#### Study setting and eligibility criteria

In order to consider GPs’ and patients’ needs alike, both GPs managing patients with CNCP and patients with CNCP themselves aged ≥ 18 years who have received opioid therapy for at least 3 months will be involved in the development of the intervention. As interviews and workshops will be conducted in German, all participants have to possess sufficient German language skills.

#### Sample size and recruitment

A minimum of six GPs and six patients with CNCP will be recruited for the interviews. The interviews will be carried out following the concept of information power until we have sufficient information power for the analysis of our research questions as well as for the generated quality of the dialog [46].

GPs will personally be invited to participate in an interview on their needs to adequately manage patients with CNCP, when they come to attend training sessions for general practice teams offered monthly at Witten/Herdecke University. Additionally, an invitation letter will be distributed via email to practices associated with the Institute of General Practice and Primary Care, Witten/Herdecke University, as teaching and/or research practices.

Patients with CNCP will be approached via teaching and research practices associated with the university Institute of General Practice and Primary Care and via patient representatives supporting the study in the GESCO advisory board.

For both GPs and patients, a balanced gender ratio will be taken into account. GPs and patients who participate in the interviews will also be asked to attend the workshop in order to support the development of the intervention.

#### Data collection methods and data management

Semi-structured interviews with GPs and patients will be conducted in person at Witten/Herdecke University, via telephone, or using a certified video conferencing system. The guidelines, which will be used for structuring the interviews, will address patients’ and GPs’ experiences regarding CNCP management, patients’ wishes related to CNCP care, and GPs’ needs for treating patients with CNCP (Supplementary Material 1 and 2). The interview guides will consider findings from the previous searches. The development will be supported by GP and patient representatives. All interviews will be conducted by researchers who are trained in qualitative methodologies. No individuals other than the interviewed person and the interviewer will be present.

In order to facilitate the analyses, all interviews will be audio-recorded and transcribed verbatim. All statements identifying a person will be anonymized in the transcription process.

#### Data analysis

In order to make the findings of the interviews promptly available for the development of the intervention, analyses of the qualitative data will be performed using rapid qualitative analysis [47]. The coding process will be responsibly managed by two researchers. The resulting themes of the qualitative analysis will be presented to stakeholders in workshops through structured reports and thematic maps. This information will guide discussions on refining and prioritizing elements of the intervention, ensuring it aligns with the qualitative insights from the study.

### Phase II: pilot testing of the GESCO intervention and the implementation strategies

#### Study design

The pilot testing of the newly developed intervention and the strategies used for implementation will be conducted as a hybrid type 2 effectiveness-implementation study and follows the guidance for conducting feasibility and pilot studies for implementation trials according to Pearson et al. This design features simultaneous testing of both (a) the feasibility of implementation and (b) clinical parameters as co-primary aims [38, 48]. In detail, an exploratory, mixed-methods study consisting of the following elements will be conducted:

- Mixed-methods process evaluation with predominantly qualitative methods in order to determine whether it is feasible to proceed to an effectiveness trial.
- Patient-centered pre-post comparison to quantify preliminary interventional effects in order to get a preview of the magnitude the intervention might have and to prepare a subsequent effectiveness trial.

#### Study setting and eligibility criteria

GPs will be eligible for the feasibility study if they manage patients with CNCP in their everyday practice and prescribe opioids for CNCP treatment. Patients will be included if they are at least 18 years old, suffer from CNCP, and have been receiving opioid therapy for at least 3 months. Patients with a clinically relevant malignant primary disease, patients currently receiving medications for opioid use disorder, and those with insufficient German language skills for participation will be excluded. A balanced gender ratio will be considered for both GPs and patients with CNCP.

#### Sample size and recruitment

Ten GPs from different general practices will be recruited for the feasibility study, excluding those practices and GPs that already participated in the development process. Again, GPs will be informed about the study and invited to participate during training sessions at the university and via email distributed to the institutes’ teaching and research practice network. Considering a patient drop-out rate of 20% and a target sample size of 40 patients for the analysis, each of the ten GPs will recruit five patients fulfilling the eligibility criteria defined for the study. This sample size is sufficient to detect, e.g., a mean difference (before and after intervention) of 0.5 on the 10-point-pain scale with a standard deviation of 1 (alpha = 0.05, power = 80%, 2-sided one-sample-*t*-test).

In order to ensure that the patients recruited for the feasibility study will match the eligibility criteria, study team will provide the GPs comprehensive written information and guidelines on how to conduct the feasibility study. To assess fidelity to the protocol, the study team will conduct study monitoring by periodic checks and also provide ongoing support to the GPs, ensuring they understand and adhere to the established procedures. This monitoring will help maintain consistency and compliance throughout the study.

#### Implementation

In order to implement the GESCO intervention in the care of patients with CNCP, the following implementation strategies [49] will be applied:

1. Educational training for GPs: Based on current knowledge GPs will take part in two educational sessions addressing
  Pharmacotherapy (targeted use of assessments, i.e., in order to screen for opioid use disorder; sex-specific pharmacotherapy; drug therapy safety)
  Strategies for patient empowerment (concepts for “de-chronification” in CNCP in consideration of the pain medications’ role for patients and their significance for coping with everyday life)
  Communication training (narrative interview techniques to facilitate a sex-, gender-, and diversity-sensitive exploration of patients; health-oriented conversation in order to promote patients’ health competencies and salutogenesis)
  Self-reflection (reflection of the GPs’ own medical actions in consideration of sex- and gender-sensitive aspects; reflection of the GPs’ individual gender role and gender awareness; introduction of mind–body approaches, i.e., stress management approaches, including practical exercises on how to instruct patients).
2. Case conferences: online quality circles for GPs to discuss care management of patients with CNCP.
3. Support: Materials and infrastructure facilitating the sustainability of the intervention in daily routine, i.e., handouts regarding communication strategies and a pharmacological hotline.

The training program will be developed in accordance with the principles for resilient learning programs of Haraldseid-Driftland and colleagues [50].

#### Intervention

The GESCO intervention comprises a sex- and gender-sensitive care concept for patients with CNCP in primary care. Applying knowledge and skills from the previous educational training, GPs will conduct two counseling sessions within 6 to 8 weeks with each of the study patients. During the counseling sessions, they are requested to obtain an expanded medical history also considering sex and gender aspects, perform a medication analysis, provide resource-oriented counseling, and—if necessary—refer the patients for psychosocial support.

The final choice of which intervention content to apply will be left to the GP’s discretion depending on the patient’s needs.

#### Outcomes

With the overall goal of determining whether a larger effectiveness trial is appropriate, this study will simultaneously test (a) the feasibility of implementing the intervention and of the trial methods and (b) the intervention’s clinical outcomes [38]:

- Implementation measures will be assessed to obtain detailed information on the implementation process and its feasibility (Table 1).
- Patient-centered clinical outcomes will be assessed to evaluate the instruments’ appropriateness and to determine preliminary effects, which will—also considering clinical relevance and results from other studies—provide the basis for an effect size estimation for a future larger effectiveness trial (Table 2).

**Table 1:** Feasibility measures based on Pearson et al. [38]

*Measure*	*Criteria*	*Data collection*	*Date of data collection*
**Implementation measures addressing the recruitment process**			
Reach	Recruitment of 10 practices for the study successful within 3 months	Documentation sheet, completed by study center	Continuously during practice recruitment and study conduct
	Success of different recruitment strategies applied for GP recruitment		
	Drop outs (GPs and patients)		
	Recruitment of 50 study patients by 10 practices successful within 3 months	Documentation sheet, completed by the practices	Continuously during patient recruitment
	Patients willingness to receive the GESCO intervention/participate in the study	Qualitative interview with GPs	After completing two consultations per study patient (t1)
**Implementation measures addressing the educational training for GPs**			
Adoption	Uptake (participation in educational trainings)	Documentation sheet, completed by study center	After educational training
Acceptability	If GPs find the intervention’s components agreeable	Evaluation sheet, completed by GPs	After educational training
Self-efficacy	Self-perceived capacity to undertake implementation		
**Implementation measures addressing the intervention on patient level**			
Fidelity	Degree to which interventional components are implemented as intended by designers (adherence)	Documentation sheet for each consultation with study patient, completed by GP	After each patient contact
Feasibility	Perceived fit of the intervention for everyday use	Qualitative interview with GPs	After completing two consultations per study patient (t1)
Adaptability	Adaptability of the intervention’s components to meet local needs		
Satisfaction	Satisfaction with the implementation strategies and intervention		
**Sustainability of implementation**			
Sustainability	Uptake of intervention	Qualitative interview with GPs incl. the G-NoMAD [53]	After completing two consultations per study patient (t1)
		Written questionnaire incl. the G-NoMAD [53], completed by GP	Simultaneously with last patient-out per practice

**Table 2:** Patient-centered clinical outcomes

*Outcome*	*Measure*	*Data collection*	*Date of data collection*		
			*t0*	*t1*	*t2*
Pain	Pain history and progression (German Pain Questionnaire [58])	Self-assessment, completed by patient	x		x
	10-point-pain scale (three scales: acute pain, average pain during the last 4 weeks, and strongest pain within the last 4 weeks)	Telephone interview with patient, conducted by study nurse	x	x	x
Mental well-being	Depression-Anxiety-Stress Scale (DASS) [59]	Self-assessment, completed by patient	x		x
	The Marburg questionnaire on habitual well-being (FW7) [60]				
Quality of life	Veterans RAND 12-Item Health Survey (VR-12) [61]				
Pain medication	Self-reported medication	Telephone interview with patient, conducted by study nurse	x	x	x
	German, nationally standardized medication plan	Assessed by treating physician	x	x	x
Adverse effects of medication	Self-reported adverse effects	Telephone interview with patient, conducted by study nurse	x		x
Satisfaction with information about medication	Satisfaction with Information about Medicines Scale (SIMS) [62, 63]				
Potential opioid medication misuse	Pain Medication Questionnaire (PMQ) [64]				
Perceived stigma due to pain	Internalized Stigma of Chronic Pain (ISCP) [65]^a^				
Disruption of daily life due to pain	Pain Disability Index (PDI) [66]				
Optimism/pessimism	Optimism–Pessimism Short Scale 2 (SOP2) [67]				

*t0* baseline (before intervention); *t1 *2-month follow-up; *t2 *5-month follow-up

^a^German translation by study team

#### Data collection and data management

Data from the participating GPs will be collected immediately after recruitment, after participation in the training, during implementation of the GESCO intervention in patient care, and after completing the intervention for all study patients (Table 1).

After recruitment, GPs will complete a written questionnaire on practice characteristics, sociodemographic characteristics, and gender awareness (Nijmegen Gender Awareness in Medicine Scale N-GAMS [50, 51]).

After participation in the educational sessions, GPs will provide written feedback regarding the sessions’ content, the materials used, the acceptability of the intervention within their practice, and their self-efficacy to apply the intervention in patient care. Also, the practices’ Organizational Readiness for Implementing Change will be assessed using the validated ORIC questionnaire [51, 52].

When implementing the intervention in CNCP care, GPs will complete a documentation sheet for each patient consultation which assesses the duration, main contents and results of the consultation, the interventional elements applied, and the GPs personal impression of the consultation. These quantitative data will be manually entered into an electronic data capture system.

For data collections with GPs that will be conducted after completing the intervention for all study patients, qualitative and quantitative methods will be applied. Immediately after finishing two consultations per study patient, a semi-structured telephone interview will be conducted with each GP (Supplementary Material 3). These interviews will be audio-recorded and transcribed afterwards. During transcription, data will be pseudonymized using a unique identification number per GP in order to link the interview data to the quantitative data assessed per physician. Accompanying the interview, the Normalization Process Theory Measure (G-NoMAD) [53, 54] will be obtained to assess the implementation process. Simultaneously with the last patient-out per practice, each GP will complete a questionnaire on sustainability aspects, which will again include the G-NoMAD [53].

In addition to the feasibility aspects assessed from the GPs, aspects on the methods’ feasibility related to the recruitment process and the implementation will be documented by the study team in order to prepare a future larger effectiveness trial (Table 1).

For patients, data will be collected three times: after enrollment (baseline, t0), about 2 months later (immediately after completing the intervention, t1), and 5 months after baseline (t2) (Table 2). All data for the patient-centered pre-post comparison, including self-reported prescribed and over-the-counter medication, will be collected from the patients via phone at baseline and after 5 months. To describe the study population sociodemographic data, gender-related variables for health research (GVHR [55], German translation by study group), and experience of social support (Oslo Social Support Scale OSSS-3 [56, 57]) will be collected at baseline. This procedure will be facilitated by a study nurse who will enter the data directly into an electronic data capture system. The data collection at 2 months will consist of a qualitative, semi-structured telephone interview and will focus on feasibility outcomes (Supplementary Material 4). In detail, the open-ended questions will address the patients’ experience with the intervention and their study participation. This includes their satisfaction with the intervention, their acceptability of the intervention, especially regarding the gender approach, and how they perceived the communication with their GP, with the study center and the data collection process. In addition to qualitative, open-ended questions, any changes in medication use since baseline will be assessed. The interview part on patients’ experiences will be audio-recorded and transcribed verbatim, whereas the data on medications will be entered directly into the electronic data capture system. Qualitative interview data will be pseudonymized using a unique identification number per patient in order to link the interview data to the quantitative data assessed for the pre-post comparison. Additionally, GPs will complete a documentation sheet for each participating patient at baseline and after 5 months, providing information on chronic diseases and prescribed medication. Medication use will be analyzed after Anatomical Therapeutic Chemical coding and in consideration of drug dosages and frequency of administration.

#### Data analysis

Analyses of the qualitative data will be performed in MAXQDA 2022 [68] using a deductive-inductive approach. To this end, deductive categories will first be defined on the basis of the interview guides. Afterwards, the coding schemes will be continuously developed and refined over time by identifying categories directly from the text material. The coding process will be responsibly managed by two researchers and will include coding sessions with a group of researchers from the GESCO study team, GP representatives, and patient representatives with CNCP.

For quantitative data, descriptive statistics will be performed using IBM SPSS Statistics for Windows [69] in order to measure preliminary effects. Patients’ baseline and follow-up data will be compared by applying a *t*-test for dependent samples, the Wilcoxon test or McNemar’s/the sign test depending on the distribution of the outcome variable. The nominal significance level for analyses will be defined as *p* < 0.05. In addition, confidence intervals will be reported for any quantities estimated.

### Patient and public involvement

The realization of the GESCO study is accompanied by a multi-perspective advisory board that includes female and male patient representatives, but also experts in general practice, pain medicine, addiction medicine, psychology, health care education, sociology, gender research, and participatory research. The advisory board members will be informed about the study process and asked for advice in regular meetings. In addition to these meetings, they will get actively involved into the following activities:

- Development of the intervention and its implementation strategy within primary care
- Preparation of the feasibility study including the development and pre-testing of study material for patients and GPs, interview guidelines, and questionnaire
- Recruitment of interview partners for the needs assessments and recruitment of primary care practices for the feasibility study
- The discussion and dissemination of results including contribution to conference presentations and to scientific or low-threshold, generally understandable publications (e.g., flyers or brochures)

In order to adequately consider the perspective of potential addressees of the GESCO intervention during the whole study, researchers working in the project planned and reflected involvement activities, which especially affect patient representatives and GPs, together with these stakeholders. For this, they used the so-called involvement matrix [70]. This process was already carried out prior to the beginning of phase I and was facilitated by an advisory board member familiar with applying the involvement matrix. It aimed to enable everyone to specify how intensively and in which project phases they would like to get involved or not to get involved.

## Ethics and dissemination

### Ethics approval

The study obtained ethical approval from the Ethics Commission of Witten/Herdecke University (reference number: 138/2022, date of approval: 08/25/2022, amendment: 08/29/2023).

### Dissemination policy

As is customary, it is planned to publish a description of the intervention components and the results of the pilot testing in international journals and to present all results at scientific conferences. In order to also make the study results transparent and comprehensible for the non-scientific public, a GESCO symposium addressing GPs, patients, researchers, and the public will be conducted at the end of the project. For this, the study conduct and its results will be prepared in simple language, which will be facilitated by patient representatives, GPs, and other members of the GESCO advisory board. Beside the public symposium, it is planned to disseminate the results at a low threshold level, i.e., via magazines of self-help organizations. The dissemination strategy will be planned together with patient representatives and GPs.

The dissemination policy will also include an analysis of whether the sex- and gender-sensitive concept developed for CNCP management might be transferable to other health care scenarios.

As the project is part of a larger funding initiative of the German Federal Ministry of Health, which aims to investigate and establish gender equality in health, information on the GESCO project and its results will also be published on the ministry’s website.

## Discussion

The GESCO study will examine how the diversity of the experience and processing of pain in patients on LTOT of differing sex and gender can be addressed appropriately and in a quality-enhancing manner in the therapeutic setting. As a result, it will provide a novel personalized concept for the care of patients with CNCP, integrated into preliminary analyses and a subsequent feasibility assessment to ascertain the suitability and implementability of the intervention. It will also be used to pilot the study instruments and measures. Our results regarding the implementation measures will be evaluated to determine the suitability of the intervention for transfer to an efficacy trial. If necessary, the intervention will be adapted or (in the worst case) rejected. The progression criteria, as outlined by Thabane and Lancaster [41] and suggested by Pearson et al. [38], will be used to guide this assessment. The limited sample size of the GESCO study restricts the generalizability of possible interventional effects but the results of this study build a foundation to estimate the sample size for a subsequent cluster randomized controlled trial taking into account the standard deviation. Additionally our estimation will be complemented by other studies from the literature applying the same outcome measures.

By considering sex and gender differences in health care and proposing a sex- and gender-sensitive care concept, the GESCO study team is doing pioneering work, which might also be transferable to other health care scenarios and thereby help improve disease management.

## Supplementary Information

Supplementary Material 1.

Supplementary Material 2.

Supplementary Material 3.

Supplementary Material 4.

## Data Availability

Not applicable.

## Abbreviations

CNCP: Chronic non-cancer pain
DASS: Depression-Anxiety-Stress Scale
FW7: The Marburg questionnaire on habitual well-being
GESCO: Gender-sensitive care for chronic non-cancer pain patients receiving long-term opioid therapy
GVHR: Gender-related variables for health research
GP: General practitioner
ISCP: Internalized Stigma of Chronic Pain
LTOT: Long-term opioid therapy
MRC: UK Medical Research Council
N-GAMS: Nijmegen Gender Awareness in Medicine Scale
NoMAD: Normalization Process Theory Measure
ORIC: Organizational Readiness for Implementing Change
OSSS-3: Oslo Social Support Scale
PDI: Pain Disability Index
PMQ: Pain Medication Questionnaire
SIMS: Satisfaction with Information about Medicines Scale
SOP2: Optimism-Pessimism Short Scale-2
VR-12: Veterans RAND 12-Item Health Survey
