# Supplementary material for: Development and feasibility of a sex- and gender-sensitive primary care intervention for patients with chronic non-cancer pain receiving long-term opioid therapy (GESCO): a study protocol
Source: Pilot Feasibility Stud. 2024 Nov 1;10:132. doi: 10.1186/s40814-024-01564-7 (PMC11529428; doi:10.1186/s40814-024-01564-7)
Supplement: Supplementary file 1 — Supplementary Material 1. [file 40814_2024_1564_MOESM1_ESM.docx]

**Needs Assessment: Interview Guideline for Patients for Assessing Needs in the Care of Chronic Pain in General Practice within the GESCO Study** (translated short Version from German Language)

| **Research Questions** | **Guideline Questions** |
| --- | --- |
| 1. Biographical/Everyday Experiences with Pain | |
| What role does the chronic pain and pain management have in the biography?  What attitudes and knowledge exist about the pain condition? | Please recall how the pain started. Can you tell me about it?   - What influence does the pain have on your everyday life? - How does your close environment deal with your pain? - What experiences have you had with (opioid) therapy changes? And with side effects? - Do you feel that you have your pain under control? - How hopeful are you that it will get better? |
| 2. Experiences with General Practitioner Care for Pain | |
| What experiences have been made with primary care treatment? | Please think about the experiences you have had with general practitioner regarding treatment of your pain. Can you tell me about it?   - How do you perceive the communication? - Do you feel that your sex/gender influences your treatment? |
| Subtopic: Barriers and Facilitators for pain management except medication | - Have you already received other prescriptions, therapies, or recommendations besides medications? - Have you tried other things outside of general practitioner prescriptions? Have you tried that? (e.g., physiotherapy, psychotherapy, yoga, meditation) - What restricts you in choosing therapies/strategies? |
| 3. Wishes and Needs for Pain Management in General Practice | |
| What wishes and needs do have patients for pain management in general practice? | - When you think about your personal treatment history: What would you wish for from your general practitioner? |
