# Supplementary material for: Development and feasibility of a sex- and gender-sensitive primary care intervention for patients with chronic non-cancer pain receiving long-term opioid therapy (GESCO): a study protocol
Source: Pilot Feasibility Stud. 2024 Nov 1;10:132. doi: 10.1186/s40814-024-01564-7 (PMC11529428; doi:10.1186/s40814-024-01564-7)
Supplement: Supplementary file 2 — Supplementary Material 2. [file 40814_2024_1564_MOESM2_ESM.docx]

**Needs Assessment: Interview Guideline for General Practitioners for Assessing Needs in the Care of Chronic Pain in General Practice within the GESCO Study** (translated short Version from German Language)

| **Research Questions** | **Guideline Questions** |
| --- | --- |
| 1. Introduction: Experience / Challenges in the Care of Patients | |
| What experiences have been made in the care of patients with chronic pain? What challenges arise? | - Think of a specific patient from your practice with chronic non-tumor pain under opioid therapy. Please tell me about him/her. - What are the challenges/difficulties from your perspective with this patient (or similar ones)? - What approach has proven effective? What tips do you have? |
| 2. Expectations, Communication, Gender/Sex | |
| What role do patient characteristics play in the treatment/communication of chronic pain? | - What expectations are placed on you by this patient? How do you handle them? - What expectations are generally placed on you by chronic pain patients? - Do people also differ in how they perceive and present pain, e.g., men differently than women? (If applicable, ask about a typical male/female case) - You also encounter patients as a man/woman. Does that influence the handling/communication? |
| 3. Therapy | |
| What experiences have been made with opioid therapy? | - What experiences have you had with this patient (if mentioned before) with Therapy with opioids and other painkillers? - What are your experiences when you want to reduce or discontinue opioids? (Do you have an example?) - Non-medication therapies: What experiences have you had with non-medication therapies (in your own practice or offered by others)? How are the therapies received? |
| 4. Wishes and Needs for Pain Management in General Practice | |
| What wishes and needs do general practitioners express for the care of patients with chronic pain?  What information/training needs exist?  How can doctors be supported/improve collaboration with other professionals? | - If you could wish for something to improve the care of chronic pain patients, what would it be? - Do you feel sufficiently informed about pain therapy? - Do you feel sufficiently informed about gender differences in pain therapy? - What information do you need to treat optimally? What is missing? (e.g., information on medication, prescriptions from other doctors, treatment of comorbidities) - How should the information ideally be provided? - What exchange with other professionals outside your practice would you like? For example, pain therapists, orthopedists... |
