# Supplementary material for: Development and feasibility of a sex- and gender-sensitive primary care intervention for patients with chronic non-cancer pain receiving long-term opioid therapy (GESCO): a study protocol
Source: Pilot Feasibility Stud. 2024 Nov 1;10:132. doi: 10.1186/s40814-024-01564-7 (PMC11529428; doi:10.1186/s40814-024-01564-7)
Supplement: Supplementary file 3 — Supplementary Material 3. [file 40814_2024_1564_MOESM3_ESM.docx]

**Guideline for General Practitioner Interviews to Evaluate the GESCO Intervention Regarding Selected Feasibility Criteria** (translated short Version from German Language)

| **Research Questions / Feasibility Criteria** | **Guideline Questions** |
| --- | --- |
| Motivation for participation / Introduction of the project in the medical practice | - You participated in the GESCO study. How was it for you? - What is the GESCO intervention for you personally? - What was your motivation to participate? - How was the GESCO study implemented in the practice team? Please describe how you approached it. |
| Educational Training (GP Level) | - When you think back to our educational training: To what extent did you feel sufficiently prepared for working with GESCO? - What was particularly helpful for you personally? - What has changed for you personally after attending the training? |
| Reach^1^  Adaptability^1^  Practicability | - Can you describe how you worked with the GESCO intervention? (How were patients selected/approached?   How did the conversations proceed?)   - Specific questions about GESCO tools presented in the educational training (as part of the intervention) |
| Medication | - How do you feel about prescribing opioids? - How has this feeling changed through your participation in GESCO? - To what extent has your attitude towards gender differences in pharmacotherapy changed during the project? |
| Satisfaction^1^ Outcome (intended/unintended) | - What changes have you noticed in your patients? - What changes have you noticed in communication with your patients? - What changes have you noticed in yourself? - How has GESCO affected your job satisfaction? - What is special about general practice in pain therapy for you? |
| Sustainability^1^ | - If you could change something about GESCO, what would it be? - What would you do differently if you were starting over? - What support would you have wished for in preparing or implementing GESCO? - How has your perception of gender in daily work changed? (Regarding yourself and/or the patients) - For which other patient groups could care like in GESCO be beneficial? |

*1: Feasibility Criteria according to Pearson et al. 2020*
