# Supplementary material for: Development and feasibility of a sex- and gender-sensitive primary care intervention for patients with chronic non-cancer pain receiving long-term opioid therapy (GESCO): a study protocol
Source: Pilot Feasibility Stud. 2024 Nov 1;10:132. doi: 10.1186/s40814-024-01564-7 (PMC11529428; doi:10.1186/s40814-024-01564-7)
Supplement: Supplementary file 4 — Supplementary Material 4. [file 40814_2024_1564_MOESM4_ESM.docx]

**Guideline for Interviews with patients to evaluate the GESCO Intervention** (translated short Version from German Language)

| **Research Questions / Feasibility Criteria** | **Guideline Questions** |
| --- | --- |
| Open Introduction | You are participating in the GESCO study on pain therapy. Can you tell me: How do you experience the participation? |
| Perception of the Intervention/Gender-Sensitive Care  Fidelity^1^  Satisfaction^1^ | - How did you perceive the conversations with your general practitioner? - Did you jointly formulate goals? What were they? - What was noted about your medications? Was anything discussed regarding opioids? Are you satisfied with your medications? |
| Improved Quality of Life  Satisfaction^1^ | - We are interested in the role the study participation and the conversation appointments played in your daily life. Can you describe what changes you noticed in your daily life? - Have your pain levels changed? - How does your environment deal with your pain? Were there any changes? - Do you feel that you are seen/heard/judged differently as a person with chronic pain? - Can you live your life the way you want? Why or why not? |
| Perception of (Gender-Sensitive) Communication  Satisfaction^1^ | - When you think about the conversations during appointments in your practice with (insert name of doctor), how did you perceive the communication? - Did you feel understood? How could you tell that you were understood? - It is often the case in life that sex/gender matters. What would you say, what role did your sex/gender play? Can you give me an example? - Do you feel that it mattered that you were treated by (insert gender identity of the treating person)? - As (insert gender identity), do you feel that your pain is treated differently in the healthcare system overall? How did you experience this in the conversations with (insert gender identity of the treating person)? |
| Perception of Data Collection | - Specific questions about data collection in the study |
| Acceptance of GESCO  Feasibility^1^  Sustainability^1^ | - What do you think we need to know to understand how the care of chronic pain can work well? - If you could change something about the GESCO approach to make it more helpful, what would that be? |

*1: Feasibility Criteria according to Pearson et al. 2020*
